# NP, OP and Derivatives in Freshwater Sediment Downstream of Textile Associated Municipal Wastewater Discharges

**DOI:** 10.1007/s00244-024-01066-w

**Published:** 2024-05-22

**Authors:** Benoit Lalonde, Christine Garron

**Affiliations:** Water Quality Monitoring and Surveillance Division, Science and Technology Branch, Environment and Climate Change Canada, 45 Alderney Dr, Dartmouth, NS B2Y2N6 Canada

## Abstract

Alkylphenol ethoxylates comprise of many anthropogenic chemicals such as nonylphenol (NP), octylphenol (OP) and nonylphenol ethoxylates (NPEOs). The objectives of this study were to assess the frequency and magnitude of detections of 4-NP, OP and NPEOs in Canadian sediment downstream of textile associated municipal wastewater treatment plants (MWWTPs) to determine if regulatory actions have had a beneficial impact on the receiving environment. Surficial sediments were obtained in four locations in the province of Québec (Canada) and were analyzed for nonylphenol, nonylphenol monoethoxylates (NP_1_EO), nonylphenol diethoxylates (NP_2_EO) and octylphenol from 2015 to 2018. Individual concentrations of the compounds varied from non detect to 419 ng/g. Of the four compounds analyzed, NP was detected the most frequently with a 75% detection rate while OPs were not detected in any of the samples. Since the Canadian regulatory actions have drastically reduced NP/NPEOs usage in textile mill factories and manufactured products, the potential source of these compounds in sediment for this study could stem from the outfall from the MWWTPs but not related to textile mills as well as from the usage of these compounds as formulants in pesticide products. Lastly, there were no exceedances to the Canadian Sediment Quality guideline toxic equivalency approach (TEQ) of 1400 ng/g or the 1310 ng/g guideline for NP in freshwater sediment from the European Scientific Committee on Health, Environmental and Emerging Risks. We hypothesize that the significant concentrations of these compounds in sediment may be a relevant and continuous source of 4NP in surface waters due to resuspension of sediment in the water column.

Nonylphenol ethoxylates (NPEOs) are a broad chemical class of compounds often referred as alkylphenol ethoxylates. Nonylphenol (NP) is probably the better-known chemical of this class and is a degradation product of NPEOs (Crane 2019). Nonylphenols are a broad category of isomeric compounds each consisting of a nine-carbon alkyl chain attached to a phenol ring, with the chemical formula C_15_H_25_O (CCME 2002). NP and NPEOs have been in use since the 1960s and are used as detergents, degreasers, wetting agents, dispersing agents, paints, and emulsifiers in various industrial sectors such as steel manufacturing, pest control products, power generation, pulp and paper processing, institutional cleaning products and textile processing (EC and HC 2001). In particular, 4-NP (Fig. 1) in the receiving environment can be attributed to breakdown from pesticide surfactant, long range transport and degradation from tire rubber (Ashley et al. 2003; Kalmykova et al. 2013; Lyons et al. 2019). In addition, octylphenol and nonylphenol polyethoxylates have been used as pesticide adjuvants with over 1000 tons NPE used in Canada for emulsification in pesticides (Bennie et al. 1997; PMRA 2010). The main areas of use of octylphenol (OP) (Fig. 1) are as an intermediate in the production of phenol/formaldehyde resins (98% of use) and in the manufacture of octylphenol ethoxylates (2% use) (OSPAR 2006).

**Fig. 1:**
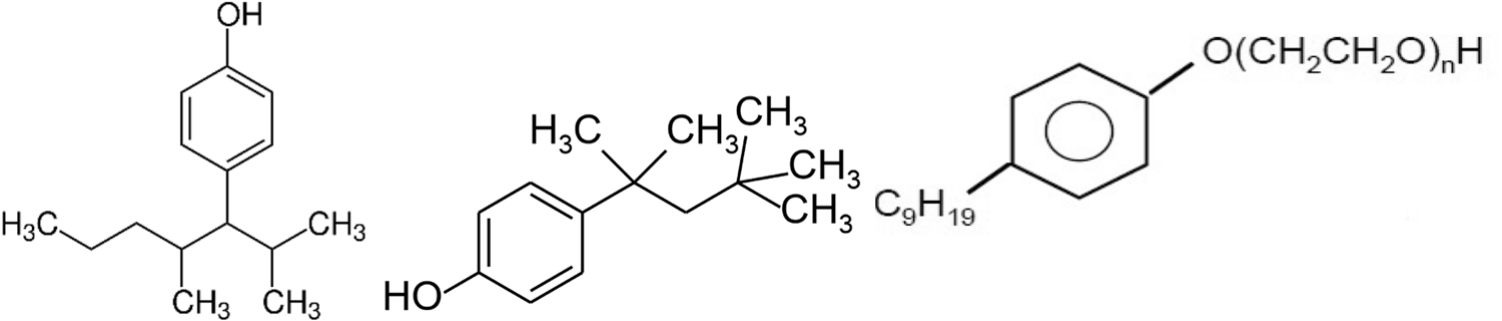
Chemical structures of 4-NP and 4-tert OP and NPEOs (www.wikipedia.com 2024)

NP, OP and NPEOs are not produced naturally and their presence in the environment is, therefore, exclusively a consequence of anthropogenic emissions (EC and HC 2001) and transformation from parent compounds (ECCC 2022). Wastewater treatment, landfill and sewage sludge recycling were the three top contributors of NP and derivatives according to Soares et al. (2008). Releases of NP, OP and NPEOs to the environment can occur at various points in the product life cycle—such as, during primary production of NPEOs or OPEs, manufacture of NPE-containing products, product use and disposal of the product to landfill, septic systems or wastewater treatment plants (EC and HC 2001). Hong et al. (2020) as well as Thiele et al. (1997) also referred to agricultural runoff as a source of nonylphenol in the water ecosystem. In general terms, alkylphenol ethoxylates (APEO) discharged to wastewater treatment plants will undergo a biodegradation thus creating biorefractory metabolites (Thiele et al. 1997). The biodegradation of the APEO usually entails a shortening of the hydrophilic chain and thus creates various metabolites such as NP, NPnEO and NPnEC (ethoxycarboxylates homologues) (Thiele et al. 1997). These resulting compounds are usually more persistent, more lipophilic and toxic than the parent compound (Thiele et al. 1997; Acir and Guenther 2018). Lastly the APEO metabolites are a complete mixtures of isomers and enantiomers with different estrogenic potential (Acir and Guenther 2018) as well as very different degradability potential by bacteria (*Sphingomonas xenophaga)* (Gabriel et al. 2005).

Sediment-water partition coefficient suggests that NP/NPEOs partition more to the particulate or sediment phase than dissolved (Hu et al. 2019; Mayer et al. 2007; Soares et al. 2008; Thiele et al. 1997). NPs also have log Kow in 4–5 range and may become bound to suspended particles especially those having high organic carbon contents (Ashley et al. 2003; Hu et al. 2019). Total and dissolved organic carbon factors can also control the dynamic distribution of NP/NPEOs (Hu et al. 2019). Due to the low water solubility of NP, OP and NPnEO and their log Kow of more than 4, sediments may be heavier contaminated by these metabolites than the surrounding water (Thiele et al. 1997). In addition, Lara-Martin et al. (2012) also mentions that NPEOs have a low biodegradation rates in sediments, especially under anaerobic conditions (Acir and Guenther 2018). Brunner et al. (1988) also mentioned that the results of the anaerobic sludge digestion of NPnEO are NPs.

Globally there are many jurisdictions that have regulated the usage of nonylphenols in order to reduce its impact to the receiving environment. Water quality regulations and/or guidelines have been developed by many jurisdictions such as the United States Environmental Protection Agency, K-Reach (Korea), Europe (under Annex XVII of REACH), Japan and the Rotterdam Convention for severely restricted compounds. However much less oversight has been afforded to creating sediment guidelines. The Government of Canada (GOC) deemed NP and NPEOs as toxic under Sect. 64 of the *Canadian Environmental Protection Act* (CEPA) 1999 (EC and HC 2001). NP, OP and NPEOs typically occur together in the environment therefore Canadian sediment quality guidelines have been developed which consider their combined effects, with a toxic equivalency approach (TEQ). The Canadian Council of the Ministers of the Environment (CCME) sediment quality guideline for NP and its ethoxylates is 1400 ng/g (1.4 mg/kg dry weight in freshwater) expressed on a TEQ basis (CCME 2002). In addition, the European Scientific Committee on Health, Environmental and Emerging Risks (SCHEER) (2022) has endorsed a quality standard in freshwater sediment for NP of 1310 ng/g (1.31 mg/kg) while the US EPA does not possess at the current time a sediment quality criteria. In 2004, Canadian risk management strategies were developed and subsequently Pollution Prevention Planning Notices were put in place for textile mills which used wet processing (ECCC 2022). Wet processes may include such activities as scouring, neutralizing, desizing, mercerizing, carbonizing, fulling, bleaching, dyeing and printing (EC 2012). The risk management objective for textile mills was a reduction in the annual use of NP/NPEOs of 97% relative to a base year to be completed by 2009 (ECCC 2022). EC (2012) published a performance report for NP and NPEOs in textile mill effluent which stated a reduction of 99.9% of NP and NPEOs from 1998 to 2009. Furthermore and during the same time period, another Canadian risk management objective achieved was a reduction of 96% in NP and NPEOs used to manufacture products and an overall reduction of 97% in NP and NPEOs imported in products (ECCC 2022).

The objectives of this study were to assess the frequency and magnitude of detection of NP, OP and NPEOs in sediment downstream of textile associated MWWTPs to determine if regulatory actions have had a beneficial impact on the receiving environment.

## Method

Sampling occurred once a year in 2015, 2016 and 2018 at 4 sites located in the province of Québec. Characteristics of the sampling sites are listed in Table 1, including population (either in the whole watershed or only the largest urban centre upstream of each site), average MWWTP discharge, and river discharge (range or annual average, if available). All four sites were in relatively small communities, with populations of less than 6000 people. Bras St. Victor was the smallest in population and had the lowest wastewater discharge of all communities. All four had textile wet processing facilities in the area, which discharge wastewater to local municipal wastewater treatment plants (MWWTPs), although no information is available on the discharges from those textile mills to the MWWTPs. The textile industries used various base materials such as wool and polyester. All four MWWTPs used aerated lagoons to treat the wastewater. At all sites, samples were collected within 250m from the outlet of the MWWTP discharges. Twelve samples were collected in total.

**Table 1:** List of monitored sites and geographical characteristics

Sites	Latitude (DD)	Longitude (DD)	Watershed population*	Avg MWWTP discharge (m^3^/s)	Stream discharge (m^3^/s)
Berthierville	46.087	− 73.167	4091	0.087	> 5000
Rivière Blanche	46.205	− 71.902	5693	0.072	< 5–20
Rivière Chaudière	46.336	− 70.921	4722	0.068	12–1100
Bras Saint Victor	46.157	− 70.919	2509	0.043	< 5–60

*DD* decimal degrees

*Population served by the WWTP. Avg; average. Discharge is meant as the volume of water that moves over a designated point over a fixed period of time

### Sample Collection

Samples were collected in depositional areas of the rivers by using an Ekman dredge (Berthierville site only) or a stainless steel spoon in case of shallow waters. Only the top 3cm were composited for analysis. We recognize that these sampling methods may introduce bias in the depositional pattern of sediment at the individual sites. Samplers used clean polyethylene gloves during the sampling procedure. All samples were collected in 60 mL laboratory certified clean amber glass jars (OSWER Directive 9240.0-05A), which were kept on ice and then frozen until delivered to the analytical laboratory. Field measurements of water temperature, conductivity, dissolved oxygen, and pH were made by a recently calibrated (less than 5 days) water quality sonde.

### Laboratory Analysis

Laboratory analyses were conducted by AXYS Analytical Services Ltd. in Sidney, BC, according to AXYS method MLA-004 v4 (AXYS 2017). Samples were spiked with ^13^C-labelled surrogate standards (^13^C_6_-4-nonylphenol and ^13^C_6_-4-nonylphenol diethoxylate) prior to extraction. Calibration standards for 4-NP and 4-*n*-OP were purchased from The Laboratory of Dr Ehrenstorfer-Schäfers, Ausburg Germany while the NP_1_EO and NP_2_EO standards were purchased from Accustandard, New Haven, USA. The surrogate standards for ^13^C_6_-4-NP and ^13^C_6_-4-NP_2_EO were purchased from Toronto Research Chemicals Inc, Toronto, Canada.

Sampled were extracted with hexane and followed by non aqueous acetylation steps (with acetic anhydride and pyridine) and were clean by column chromatography on a 28% deactivated basic silica column (AXYS 2017). After cleanup and before instrumental analysis recovery, a labeled recovery standard (i.e., d_10_-pyrene) is added. The typical final extract volume is 500 µL. The extract was analyzed on a Restek™ Rtx™-5 capillary gas chromatography column coupled to a low-resolution mass spectrometer (LRMS) (AXYS 2017).

The LRMS is operated at a unit mass resolution in the electron ionization mode using multiple on detection acquiring at least two characteristics ions for each target analyte and surrogate standard (AXYS 2017). Target analytes were detected using multiple ion detection with acquisition of at least two characteristics ions for each target analyte and stable isotope internal standard (AXYS 2017). Initial calibration is performed using a six-point calibration series of solutions that encompass the working concentration range. Typical concentration of calibration standards varied from 150 to 75,000 ng/mL (AXYS 2017). The concentrations of NPs and derivatives were quantified as the sum of individual marker peaks in the GC/LRMS chromatogram to yield total amounts while OP was quantified on one peak. Quantification ions used for each of the four analytes are listed in the supplementary information of Klosterhaus et al. (2013). All analysis were done on an Agilent 5973 Network Mass Selective Detectors equipped with an Agilent 6890N Network Gas Chromatograph, a CTC GC-PAL series autosampler and a PC workstation running ChemStation software.

Sample specific detection limit is determined individually for every sample analysis run by converting the area equivalent of 3.0 times the estimated chromatographic noise height to a concentration in the same manner that target peak responses are converted to final concentrations. Sample specific detection limits for NP, OP and derivatives varied between sample batches but ranged from 0.12 to 3.84 ng/g (dry weight) and averaged 1.06 ng/g.

All samples were frozen and then analyzed at AXYS during the same time period and as such only one set of laboratory blanks was processed. All laboratory blanks had concentrations below the detection limit of the laboratory. The percent recoveries, based on a spiked matrix sample (AXYS 2017), were between 70 and 130% and were deemed acceptable.

### Statistical Analysis

All statistical tests and graphs were produced with Systat™13 and R (2013). Depending on the compounds, a large portion of the dataset was below the detection limit (DL) of the laboratory. We acknowledge that there are packages in R that deal specifically with datasets containing values below the detection limit such as NADA or NADA2 which we have used extensively in the past (Lalonde and Garron 2021). However, the proportion of censored values in our dataset precludes us from using these packages properly (Antweiler 2015). Therefore we omitted all values under the DL from the summary statistics and figures presented in this study.

## Results and Discussion

### Geographical Distribution

**Fig. 2:**
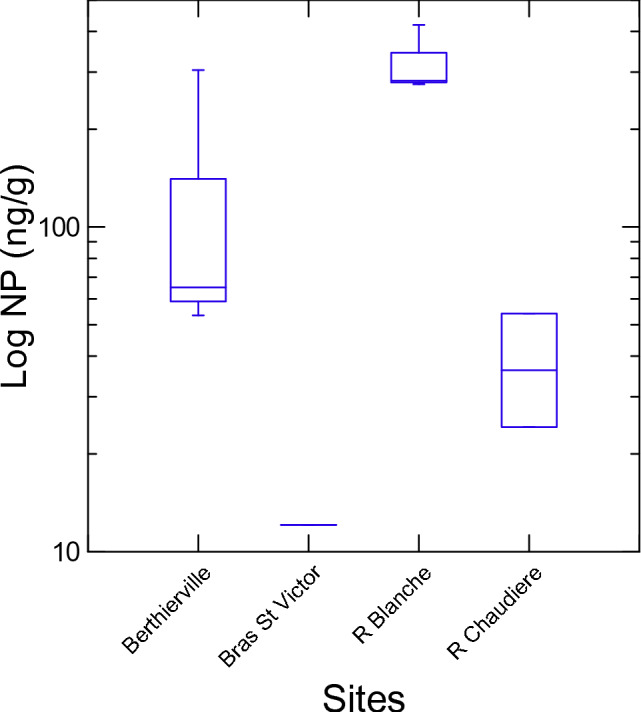
Box plots of NP concentrations (ng/g dry weight) on a logarithmic scale as a function of sampling sites. The median is represented by the line in the middle of the boxes, while the first and third quartile are the end of both sides of the boxes. The minimum and maximum values are represented at the tip of each of the lines

Nonylphenol was detected in the majority of samples with only 3 out of the 12 samples declared non-detects. NP concentrations ranged from 12.1 (2015—Bras St Victor) to 419 ng/g (2018-Rivière Blanche) (Fig. 2) NP_1_EO was detected in half of the samples. It was measured at Berthierville and Rivière Blanche in 2015, 2016 and 2018 but was undetected at the other two sites. NP_1_EO concentrations ranged from 1.95 to 81.8 ng/g (2018) with the highest values of 49.7 and 81.8 ng/g measured in the Rivière Blanche. NP_2_EO was detected only two times and only at the Rivière Blanche sampling site with concentrations of 7.54 and 7.06 ng/g, respectively, in 2015 and 2016. OP concentrations were all below detection for all years and at all sites, which is similar to Pignotti and Dinelli (2018) and Crane (2019) who did not detect any OP in freshwater sediments in Italy and in the United States, respectively. Out of the four compounds analyzed in this study, NP is the dominant AP compound detected, which is very similar to the studies from Mayer et al. (2007), Crane (2019) and Xie et al. (2020) with all three citing 4-NP as the dominant component of APs in sediment. The breakdown of NPEOs to NP is well established (Crane 2019) and since both Rivière Blanche and Berthierville had detectable concentration of NPEOs, it is not surprising that these two sites had the highest concentrations of NP in this study.

### Temporal Variation

**Fig. 3:**
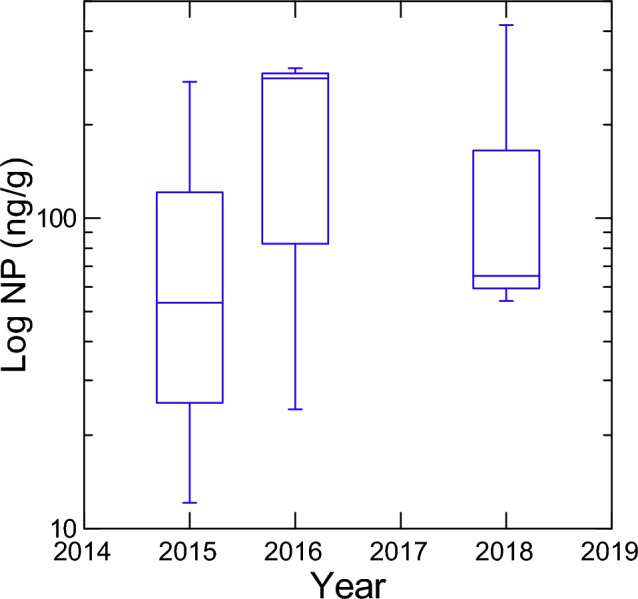
Box plots of NP concentrations (ng/g dry weight) on a logarithmic scale as a function of sampling years. The median is represented by the line in the middle of the boxes, while the first and third quartile are the end of both sides of the boxes. The minimum and maximum values are represented at the tip of each of the lines

As for the concentrations of NP over the 3 years of the study, the median was highest in 2016 compared to 2015 and 2018 (Fig. 3), which indicates no obvious increasing or decreasing trend over that time. Since concentrations of NP/ NPEOs did not decrease with time at our sampling site, we hypothesize that there are other potential source of alkylphenols in these watersheds that are not associated with textile mill effluent. The half life in marine sediments was calculated at over 60 years but this is probably due to lower temperatures and lack of oxygen (Shang et al. 1999) compared to our freshwater sediment sites of this study. However, the surficial sediments obtained for this study were in an oxic environment and since the half life of NP is 66 days (based on a freshwater mesocosm study—Heinis et al. 1999), it seems likely that the compounds were subject to degradation. Therefore, the concentrations measured as part of this study do not stem from legacy NP/NPEOs from the textile mills located in these watersheds but rather from regular new inputs of NP/NPEOs at these sites.

From the frequency of detection, it appears that the Rivière Blanche sampling site is the most impacted by NP and NPEOs in our study. NP/ NPEOs concentrations in water have been analyzed a few times at two of our sites (Rivière Blanche and Bras St Victor). Interestingly, St Victor in 1991 had an NP concentration of 3300 ng/L which diminished to < 100ng/l in 2010 and to a median of 0.4 ng/L in 2014–2019) (Berryman et al. 2012; Lalonde and Garron 2021). In addition, the Rivière Blanche site had an NP concentration of 100 ng/L which diminished slightly to a median of 58.4 ng/L in 2014–2019 (Berryman et al. 2012; Lalonde and Garron 2021). Both sites had no NP_1_EO or NP_2_EO detections in 2010 while only Rivière Blanche had detections in the years of this study. A drastic reduction (96–99%) of NP/NPEOs in textile mill effluents as well as in manufactured products in Canada has been established since at least 2009 (EC 2012; ECCC 2022). Therefore, if the textile industries in these communities have, in fact, ceased use of NP compounds in response to regulatory requirements in Canada, the NP compounds measured in this study are likely attributed, at least in part, to other sources. Those could be either the discharge of household products to wastewater treatment plants, wash off from roads contributing residue from tires, or potentially from runoff from agricultural pesticide applications in in the area. Whatever the source, we can deduce that concentrations measured in this study were not entirely legacy residues from textile activities.

### Comparison to TEQs

NP, OP and NPEOs typically occur together in the environment therefore guidelines have been developed which consider their combined effects, with a toxic equivalency approach (TEQ). CCME sediment quality guideline for NP and its ethoxylates is 1400 ng/g (1.4 mg/kg) expressed on a TEQ basis (CCME 2002). Toxic equivalency factors (TEF) used in the TEQ calculations have values of 1, 0.5 and 1 for NP, NP*n*EO (1 ≤ *n* ≤ 8) and OP respectively (CCME 2002).

In comparison to the TEQ, the Rivière Blanche site has the highest total of all sites samples and is approximately 1/3 of the concentration at which ecological harm may occur (Table 2). Furthermore, the 2018 results at Rivière Blanche were the highest TEQ in all 3 years of sampling (Table 2). Based on the results of this study, it appears that the sediment concentrations of NP, OP and derivatives were not of concern at those particular sites at the time of this study. The only Canada wide study demonstrated that in 2015–2016 out of 27 sites sampled, 24 had detection of at least one NP or NPEOs in freshwater sediments (ECCC 2022). One site (Still Creek in British Columbia) even had a TEQ value (5879 ng/g) exceeding the guideline value of 1400 ng/g (ECCC 2022).

**Table 2:** TEQ calculations for the sampling sites by years (ng/g)

Sites	2015	2016	2018
Berthierville	56	312.8	66.1
Rivière Blanche	319	291.8	443.8
Rivière Chaudière	0	24.2	54.1
Bras St Victor	12.1	0	0

### Comparison to Other Canadian and International Studies

Over the last few decades there was a large effort to determine NP/NPEOs concentrations in surface water both in Canada and internationally. Studies to determine concentrations in sediments are more sporadic. However, NP/NPEOs discharged into the aquatic environment have a higher association with sediments rather than the dissolved phase (Soares et al. 2008).

Table 3 below presents some of those sediment studies both in Canada and internationally. In Canada only two studies with published sediment concentrations were located. Both studies were conducted before any regulatory mechanism to decrease NP/NPEOs in the environment were enacted. Therefore, the maximum concentrations obtained by Mayer et al. (2007) and Bennie et al. (1997) are at least one or two orders of magnitude higher than our study. In the United States, the Crane (2019) study measured concentrations above those of this study but similarly to our results, no OP compounds were detected in sediments. Crane (2019) suggested that the lack of OP in sediment was due to an overwhelming usage of NPEs in comparison to octylphenol ethoxylates as well as the enhanced hydrophobicity of NP over OPs.

**Table 3:** Concentration range or individual values of alkylphenols in Canadian and international studies

Sources	NP (ng/g)	NP_1_EO (ng/g)	NP_2_EO (ng/g)	OP (ng/g)	References
*Canada*					
Cootes Paradise, Ontario	Nd–1750	Nd–1250	Nd–690	Nd–52	Mayer et al. (2007)
Great Lakes, upper St Lawrence R., Ontario	170–72000	Nd–38000	Nd–6000	Nd–1800	Bennie et al. (1997)
*International*					
Stormwater ponds—Minnesota, USA (mean)	7611	688.5	158	nd	Crane (2019)
Delaware and Schuylkill Rivers, USA	140–12000	na	na	na	Ashley et al. (2003)
Rivers, California, USA	Nd–7750	na	na	na	Lyons et al. (2019)
San Fransico Bay, USA	86	40	na	na	Klosterhaus et al. (2013)
Sites across United States	20.8	12.8	179	0.789	Newsted et al. (2023)
Hanjiang River (China)	Nd–35	na	na	Nd–38	Hu et al. (2019
Rivers (Greece)	42	31	36	na	Koumaki et al. (2018)
Pearl River (China)	128–3831	na	na	4.59–79	Xie et al. (2020)
River (Italy)	Nd—97	na	na	Nd	Pignotti and Dinelli (2018)

*Nd* denotes values under the detection limit of thew laboratory. *na* denotes no attempt at measuring this particular compound

Newsted et al. (2023) published an exhaustive review of NP concentrations across the United States and concentrations for NP, NP_1_EO and NP_2_EO were similar to those of this study. The greatest contributions of APEOs to the TEQs calculated in sediments in the Newsted et al. (2023) study were from NP and NP_2_EO while the highest contributions were from NP and NP_1_EO in this study. Lastly the international studies yielded some comparable results to our own (Hu et al. 2019, Koumaki et al. 2018; Pignotti and Dinelli 2018) while one other study in the Pearl River of China (Xie et al. 2020) surpassed all the concentrations measured in this study.

Although there are a few regulations on the use and discharge of NP/NPEOs in Canada, a Canadian industrial survey performed in 2016–2017 indicated that there are still significant amounts of NP/NPEOs in manufacturing (265,575 kg), usage (3,304 657 kg) and importations (5,473,293 kg) with the majority (60%) of the amounts used by the manufacturing sector (ECCC 2022). Therefore, it is likely that one of the sources of the NP/NPEOs in sediment in this study stem from the outputs from the MWWTPs which ultimately captured those compounds from manufactured processes or products. Another possible source is the use of NP/NPEOs in pesticide products as a formulant. In 2001, it was identified that two hundred and eleven registered pesticides products contained NP or NPEOs were of current use in Canada (EC and HC 2001). In 2022, nonylphenol are still listed as an acceptable formulant in pest control products currently registered for use in Canada. Both the St Victor and Rivière Blanche sampling sites have agricultural inputs upstream of our sampling locations. However, the Rivière Blanche site (Blanche River) is a much smaller watercourse in comparison to the Bras Saint Victor and perhaps offers much less dilution capacity for both the possible agricultural (pesticide) and MWWTP inputs. This could explain the higher concentrations of NP/NPEOs detected in Rivière Blanche. Lastly, Kalmykova et al. (2013) also mentioned long range transport as a source of NP. However, this source seems unlikely to explain the results of this study as our sites are located close together and therefore would be subject to the same long range wind patterns and atmospheric depositions.

## Conclusion

None of the NP/NPEOs values measured as part of this study were close to the Canadian sediment quality guideline but for one sampling site which has concentrations of half of the guideline. Only one other international guideline (SHEER 2022) was found for freshwater sediments for the individual substance NP and none of the values in this study were above this guideline either.

Interestingly, it is hypothesized that the source of NP/NPEOs in sediment has shifted since the enaction of the pollution reduction measures for textile mills and manufactured products. Since the textile mills achieved a reduction of over 99% of NP/NPEOs in their effluent, the concentrations of NP/ NPEOs detected during this study are likely from other sources than those textile mills located at each sampling sites. Although the purpose of this study was not to find the source of NP/NPEOs, it seems likely that the compounds are from the discharge of the MWWTP from domestic uses, road run-off and possibly from pesticide formulations.

Therefore, it is suggested that sediment sampling campaigns be established to be able to detect these important but overlooked sources of alkylphenols in the Canadian freshwater environment as the compounds in sediments may serve as a source in surface water when the sediments are re-suspended following weather events such as flooding or dredging activities (ECHA 2013).

## Data Availability

The datasets generated during and/or analyzed during the current study are available from the corresponding author on reasonable request.
